# Availability, price and affordability of cardiovascular medicines: *A comparison across 36 countries using WHO/HAI data*

**DOI:** 10.1186/1471-2261-10-25

**Published:** 2010-06-09

**Authors:** Maaike SM van Mourik, Alexandra Cameron, Marg Ewen, Richard O Laing

**Affiliations:** 1 Student at the Faculty of Medicine, University Medical Centre, Utrecht, The Netherlands; 2 Department of Essential Medicines and Pharmaceutical Policies, World Health Organization, Geneva, Switzerland; 3 Division of Pharmacoepidemiology & Clinical Pharmacology, Utrecht Institute for Pharmaceutical Sciences, Utrecht University, Utrecht, The Netherlands; 4 Health Action International Global, Amsterdam, The Netherlands

## Abstract

**Background:**

The global burden of cardiovascular disease (CVD) continues to rise. Successful treatment of CVD requires adequate pharmaceutical management. The aim was to examine the availability, pricing and affordability of cardiovascular medicines in developing countries using the standardized data collected according to the World Health Organization/Health Action International methodology.

**Methods:**

The following medicines were included: atenolol, captopril, hydrochlorothiazide, losartan and nifedipine. Data from 36 countries were analyzed. Outcome measures were percentage availability, price ratios to international reference prices and number of day's wages needed by the lowest-paid unskilled government worker to purchase one month of chronic treatment. Patient prices were adjusted for inflation and purchasing power, procurement prices only for inflation. Data were analyzed for both generic and originator brand products and the public and private sector and summarized by World Bank Income Groups.

**Results:**

For all measures, there was great variability across surveys. The overall availability of cardiovascular medicines was poor (mean 26.3% in public sector, 57.3% private sector). Procurement prices were very competitive in some countries, whereas others consistently paid high prices. Patient prices were generally substantially higher than international references prices; some countries, however, performed well. Chronic treatment with anti-hypertensive medication cost more than one day's wages in many cases. In particular when monotherapy is insufficient, treatment became unaffordable.

**Conclusions:**

The results of this study emphasize the need of focusing attention and financing on making chronic disease medicines accessible, in particular in the public sector. Several policy options are suggested to reach this goal.

## Background

The global burden of cardiovascular diseases (CVD) is on the rise and at present 30% of all deaths worldwide are due to cardiovascular conditions [1]. The great majority (80%) of these deaths now occur in developing countries [2]. Recent projections on the global burden of disease predict that mortality rates of cardiovascular diseases will continue to increase and CVD will rank as leading cause of death in all parts of the world by 2030 [3]. The epidemiological transition describes how changes in lifestyle and healthcare system lead to an increasing percentage of deaths due to chronic cardiovascular conditions [4]. In order to successfully treat these conditions and reach to the next stage of the epidemiological transition in which deaths are prevented and the onset of disease is delayed, adequate treatment and prevention programs need to be implemented. Within this treatment strategy, ongoing pharmaceutical management with combination therapy using multiple medicines is of great importance. Recurrences of CVD can be diminished by 75% when adequate combination therapy is used [5].

The WHO-PREMISE study investigating the secondary prevention of CVD in ten low- and middle income countries found that pharmaceutical use was often insufficient. Among the patients with coronary heart disease, 18.8% did not receive aspirin, 51.9% did not receive a beta-blocker, 60.2% did not receive an angiotensin-converting enzyme (ACE) inhibitor and 79.2% did not receive a statin [6]. Whether this lack of pharmaceutical treatment was due to non-compliance or limited access to medicines was not investigated in this study, however these results call for further research.

In the past, a number of studies on medicine availability and prices have been undertaken, some of which focused specifically on cardiovascular medicines. A Mexican study found that hydrochlorothiazide was the only treatment option that did not become less affordable between 1990 and 1996; 1.1% of the minimum wage was needed to pay for chronic treatment. Other CVD medicines increased up to threefold in price and treatment cost as much as 47% of the minimum wage [7]. A study performed in Ghana found that 93% of patients did not comply with their antihypertensive treatment regimen, 96% of which reported high medicine prices as the reason for not complying [8]. Other studies investigating medicine pricing in general, including cardiovascular medicines, found that medicines were generally poorly available and highly priced [9-12].

Many of the investigations, however, did not use a standardized methodology, which makes it difficult to compare results across studies. In order to facilitate more standardized research into medicine prices, Health Action International (HAI) and the World Health Organization (WHO) have developed a standardized survey methodology to investigate medicine prices, availability, affordability and price components in the supply chain. Previous analyses of WHO/HAI data for medicines used to treat chronic conditions such as hypertension, asthma and diabetes showed that medicines were poorly available, in particular in public sector facilities. Prices of medicines varied greatly across countries and the public sector was usually less expensive than the private sector, as were generics compared to originator brand products. Many treatment options were not affordable, especially when treatment with multiple medicines was necessary [13,14]. Another analysis of the data for a basket of medicines across 36 countries, which corrected for inflation and purchasing power, showed very similar results [15].

The aim of this study is to perform a secondary analysis of the availability, price and affordability of chronic-care cardiovascular medicines in developing countries using the WHO/HAI data, and correcting for inflation and purchasing power. The medicines studied are atenolol, captopril, hydrochlorothiazide, losartan and nifedipine, all of which can be used to treat hypertension.

## Methods

### WHO/HAI data collection

The WHO/HAI methodology aims to generate reliable information concerning the availability, price and affordability of medicines and is briefly summarized here. All surveys analysed in this research used the methodology described in the first edition of the manual [16].

In each survey, data on availability and patient prices are collected in a sample of outlets in the public and private sector. Public sector procurement prices are also collected, usually at a central level such as the Ministry of Health or the Central Medical Store. In each survey, one major urban centre and three randomly-chosen administrative regions within one day's travel of the urban area are selected as survey areas. In each survey area, the main public hospital and at least four other public medicine outlets are selected to obtain data on public sector patient prices. The private sector sample is chosen by selecting the private outlet closest to each sampled public sector outlet. Thus, a minimum of 20 outlets per survey per sector are sampled. In 2008, a second edition with slightly different methodology was published [17].

Surveys are performed according to a standardized protocol with results double-entered into a uniform workbook for data analysis. In each survey, data are collected on 30 (global) core medicines and up to 20 supplementary medicines of local or regional importance. For each medicine a fixed dosage form and strength is used. Surveys are usually performed on a national level, but large countries such as India and China have been surveyed on a sub-national basis.

### Outcome measures

Availability is expressed as the percentage of facilities where the medicine is found on the day of data collection. Only formulations of the same strength and dosage form are included.

Price is expressed as a price per unit (e.g. tablet, dose) and converted to a median price ration (MPR) by dividing the median local price by an international reference price (IRP). The IRP is obtained from the Management Sciences for Health International Drug Price Indicator Guide which reports median prices of high quality multi-source medicines offered to developing and middle-income countries by different suppliers [18]. Each survey uses the IRP for the year prior to the survey and the IRP is converted to local currency using the exchange rate on the first day of data collection. Price ratios are not calculated when the medicine was present in less than four outlets. In addition, treatment affordability is estimated by calculating the number of day's wages the lowest-paid unskilled government worker needs to purchase one month's supply of medicines according to a standard treatment regimen.

### Secondary data analysis

The following anti-hypertensive medicines have been included: captopril 25 mg, atenolol 50 mg, hydrochlorothiazide 25 mg, nifedipine retard 20 mg and losartan 50 mg, all in either tablet or capsule form. Out of 19 anti-hypertensive medicines in the database, these medicines were chosen because they are the most-surveyed representatives of five major pharmaceutical classes. Price information was not analysed for losartan because there was no reliable IRP available; losartan is not widely produced as a generic formulation because it is still under patent in many countries [19]. At the time of the study, 45 surveys from 36 countries were available for analysis (Table 1). The most recent data were extracted from the WHO/HAI database which is available online [20].

**Table 1:** Countries surveyed classified by World Bank income group

Income group	Countries surveyed
Low income (LI)	Chad, Ethiopia, Ghana, India (Chennai, Haryana, Karnataka, Maharashtra 12 districts, Maharashtra 4 regions, Rajasthan, West-Bengal), Kenya, Kyrgyzstan, Mali, Mongolia, Nigeria, Pakistan, Sudan (Gadarif, Khartoum, Kordofan), Tajikistan, Tanzania, Uganda, Uzbekistan, Yemen.
Lower-middle income (LMI)	Armenia, Cameroon, China (Shandong, Shanghai), El-Salvador, Fiji, Indonesia, Jordan, Morocco, Peru, Philippines, Sri Lanka, Syria, Tunisia.
Upper-middle income (UMI)	Brazil (Rio de Janeiro), Kazakhstan, Lebanon, Malaysia, South Africa (Kwazulu Natal State)
High income (HI)	Kuwait, United Arab Emirates

Abbreviations: LI - Low income; LMI - Lower middle income; UMI - Upper middle income; HI - High income.

In order to compare prices across different countries and regions, several data adjustments have been made. All prices have been converted back to the year 2004, as the majority of surveys were conducted in this year. Prices were standardized to IRPs for 2003 and to a common US dollar exchange rate as published by the International Monetary Fund [21]. For Uzbekistan the exchange rate published by the United Nations Economic Commission for Europe was used; due to the multiple exchange rates for Syria the exchange rate used in the surveys was left unchanged.

To correct for either inflation or deflation that may have occurred between the survey year and the base year (2004), prices were adjusted using the consumer price index (CPI) [21]. The consumer price index is a standardised measure of inflation which is calculated by comparing the price of a standardized basket of goods in the current year to a baseline year. Adjusting prices to represent the survey year 2004 (thus using 2003 IRPs and correcting prices using the CPI) allowed for evaluation of public procurement prices across surveys and comparison of procurement prices with public sector patient prices within surveys.

In order to compare patient prices across countries and/or regions, prices were also adjusted for purchasing power parity (PPP) [21]. PPP quantifies how much of a basket of goods a specific quantity of US dollars will buy in a specific country. Procurement prices were not corrected for PPP because the pharmaceutical procurement market is international and competitive and the price a country pays generally does not depend on the purchasing power of its inhabitants.

Brand premiums were calculated by dividing the adjusted MPR for an originator product by the adjusted MPR for its lowest-priced generic equivalent.

Data have been analysed by 2007 World Bank Income Groups (Table 1) [22]. Results are reported as averages for each income group.

Finally, in El-Salvador, India, Kuwait, Malaysia, Morocco, Pakistan, Tunisia, Uganda and the United Arab Emirates medicines were provided free-of-charge in the public sector and as such no pricing data was analyzed for the public sector in these countries.

## Results

### Availability

Availability of cardiovascular medicines varied considerably across the surveyed countries, even within income groups. Availabilities ranging from 0 to 100% within one income group were no exception (tables 2 and 3).

**Table 2:** Public sector percentage availability by World Bank income group (weighted averages).

	Atenolol		Captopril		HCT		Losartan		Nifedipine		All	
	LPG	OB	LPG	OB	LPG	OB	LPG	OB	LPG	OB	LPG	OB
	%	%	%	%	%	%	%	%	%	%	%	%
	(n)	(n)	(n)	(n)	(n)	(n)	(n)	(n)	(n)	(n)		
**LI**	40.7 (20)	0.8 (20)	18.6 (21)	1.4 (21)	15.0 (21)	0.4 (16)	2.2 (16)	0.0 (13)	24.5 (19)	0.2 (19)	20.8	0.6
**LMI**	17.8 (8)	3.8 (8)	59.4 (9)	8.7 (9)	51.3 (9)	0.0 (9)	8.6 (8)	12.1 (8)	20.4 (8)	21.5 (8)	32.6	9.0
**UMI**	5.0 (3)	3.3 (3)	5.0 (3)	66.7 (3)	33.3 (2)	0.0 (2)	0.0 (3)	30.0 (3)	35.0 (3)	0.0 (3)	14.4	21.4
**HI**	93.0 (2)	10.5 (2)	81.3 (2)	5.6 (2)	46.9 (2)	0.0 (1)	0.0 (1)	72.2 (1)	50.0 (2)	100.0 (2)	60.3	38.1

**All**	**38.9** **(33)**	**2.3** **(33)**	**31.5** **(35)**	**9.1** **(35)**	**27.7** **(34)**	**0.2** **(28)**	**3.7** **(28)**	**10.4** **(25)**	**26.0** **(32)**	**11.7** **(32)**	**26.3**	**6.8**

Abbreviations: HCT - hydrochlorothiazide; LPG - lowest priced generic; OB - originator brand; LI - Low income; LMI - Lower middle income; UMI - Upper middle income; HI - High income.

**Table 3:** Private sector percentage availability by World Bank income group (weighted averages).

	Atenolol		Captopril		HCT		Losartan		Nifedipine		All	
	LPG	OB	LPG	OB	LPG	OB	LPG	OB	LPG	OB	LPG	OB
	%	%	%	%	%	%	%	%	%	%	%	%
	(n)	(n)	(n)	(n)	(n)	(n)	(n)	(n)	(n)	(n)		
**LI**	79.7 (22)	32.5 (22)	25.9 (23)	24.0 (23)	35.5 (22)	1.7 (18)	46.0 (16)	5.7 (13)	74.8 (21)	13.0 (21)	52.3	17.0
**LMI**	59.1 (10)	38.9 (10)	83.5 (11)	39.4 (11)	64.3 (10)	8.9 (10)	37.8 (10)	42.9 (10)	45.6 (9)	38.6 (9)	58.8	33.9
**UMI**	72.3 (4)	66.8 (4)	68.5 (4)	84.4 (4)	55.5 (3)	21.7 (3)	15.0 (3)	66.7 (3)	82.1 (3)	36.9 (3)	60.1	57.7
**HI**	76.0 (2)	98.0 (2)	16.7 (2)	94.0 (2)	50.0 (2)	0.0 (1)	0.0 (1)	100 (1)	34.8 (2)	98.0 (2)	39.4	85.0

**All**	**73.3** **(38)**	**42.8** **(38)**	**59.4** **(40)**	**36.5** **(40)**	**45.9** **(37)**	**6.7** **(32)**	**38.6** **(30)**	**29.8** **(27)**	**65.6** **(35)**	**26.5** **(35)**	**57.3**	**29.2**

Abbreviations: HCT - hydrochlorothiazide; LPG - lowest priced generic; OB - originator brand; LI - Low income; LMI - Lower middle income; UMI - Upper middle income; HI - High income.

Overall, atenolol 50 mg had the highest availability for the lowest-priced generic (LPG) in both the public and private sectors (38.9% and 73.3% respectively) and losartan the lowest. The entire basket of cardiovascular medicines had an average availability of 26.3% for the LPG in the public sector and 57.3% in the private sector (LPG). For all medicines, the private sector had better availability than the public sector, both for LPGs and originator brand products (OB).

In nearly all surveys, LPGs had a higher availability than originator brand products. The only exception to this was losartan, which is still under patent; ten out of thirty surveys had better availability of the OB. Across income groups, in both the public and private sector, higher income regions tended to have better availability than lower income regions. In the private sector, higher income areas with a low availability of LPGs had a high availability of OBs.

### Public sector procurement prices

**Figure 1:**
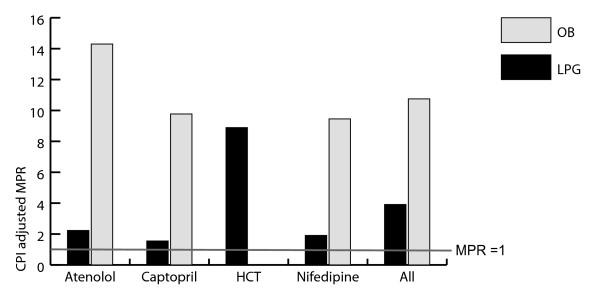
**Procurement MPRs (CPI adjusted) for the LPG and the OB**. The line represents a price ratio equal to 1.0 (procurement at the same price as the international reference price). Abbreviations: HCT - hydrochlorothiazide; LPG - lowest-priced generic; OB - originator brand; MPR - Median Price Ratio.

A comparison of public sector procurement prices showed that they varied greatly both across medicines and countries/regions. For example, the CPI-adjusted MPR for captopril was 0.21 in Peru and the United Arab Emirates and 12.75 in Morocco. This means that prices ranged from 0.21 to 12.75 times the international reference price. The average procurement MPR for both generic and originator products are summarized in Figure 1. It is important to realize that MPR is a measure relative to the international reference price, not an absolute measure. This may explain the high MPR for hydrochlorothiazide. There was no data available concerning the procurement of originator brand hydrochlorothiazide.

No particular relationship could be found across World Bank Income Groups. Nigeria and Mongolia had the highest procurement prices for several medicines. Some countries were capable of obtaining consistently low procurement prices, in particular Ethiopia, Fiji and Jordan.

Data matched by medicine, which allow for comparison of procurement prices and public sector patient prices within one survey, were only available for a very limited number of surveys and medicines (data not shown). It could be seen that in Ethiopia and Tanzania patient prices were at least 50% more expensive than the procurement price for three out of four medicines. On the other hand, patient prices were lower than procurement prices for several medicines in Nigeria.

### Patient prices

**Figure 2:**
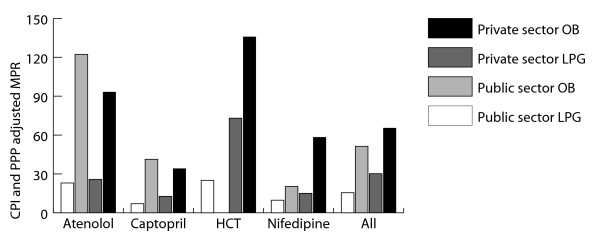
**Average CPI and PPP adjusted MPRs for atenolol, captopril, hydrochlorothiazide and nifedipine**. Abbreviations: HCT - hydrochlorothiazide; LPG - lowest priced generic; OB - originator brand; MPR - Median Price Ratio.

Figure 2 summarizes the average median price ratios for the LPG and the OB in the public and private sector. Data classified by income group is presented in tables 4 and 5.

**Table 4:** Public sector patient MPR (CPI and PPP adjusted), by World Bank income group (weighted averages).

	Atenolol		Captopril		HCT		Nifedipine		All	
	LPG	OB	LPG	OB	LPG	OB	LPG	OB	LPG	OB
	(n)	(n)	(n)	(n)	(n)	(n)	(n)	(n)		
**LI**	15.7 (7)		7.2 (7)	15.8 (1)	40.5 (4)		9.8 (6)		15.9	15.8
**LMI**	40.2 (3)	122.3 (1)	6.9 (6)	71.2 (2)	12.0 (4)		9.5 (2)	20.4 (1)	15.3	71.3
**UMI**				7.0 (1)	15.2 (1)		9.5 (1)		12.4	7.0
**HI**										

**All**	**23.0** **(10)**	**122.3** **(1)**	**7.0** **(13)**	**41.3** **(4)**	**25.0** **(9)**		**9.7** **(9)**	**20.4** **(1)**	**15.5**	**51.3**

Blank cases are due to missing data. Abbreviations: LPG - lowest priced generic; OB - originator brand; HCT - hydrochlorothiazide; LI - low income; LMI - lower-middle income; UMI - upper middle income; HI - high income.

**Table 5:** Private sector patient MPR (CPI and PPP adjusted), by World Bank income group (weighted averages).

	Atenolol		Captopril		HCT		Nifedipine		All	
	LPG	OB	LPG	OB	LPG	OB	LPG	OB	LPG	OB
	(n)	(n)	(n)	(n)	(n)	(n)	(n)	(n)		
**LI**	21.0 (22)	107.3 (13)	12.4 (21)	3.9 (9)	85.2 (16)	73.0 (1)	11.8 (20)	51.6 (7)	35.6	74.9
**LMI**	41.5 (9)	87.6 (7)	14.7 (12)	39.8 (10)	66.6 (10)		27.8 (6)	81.8 (6)	45.7	70.0
**UMI**	13.2 (3)	83.4 (3)	8.9 (3)	31.6 (4)	36.0 (3)	156.8 (3)	11.1 (3)	46.0 (2)	22.4	81.6
**HI**	26.8 (2)	37.2 (2)	10.7 (2)	12.1 (2)	55.2 (1)		13.9 (1)	23.6 (2)	38.5	31.3

**All**	**25.8** **(36)**	**93.2** **(25)**	**12.7** **(38)**	**34.1** **(25)**	**73.0** **(30)**	**135.8** **(4)**	**15.0** **(30)**	**58.3** **(17)**	**30.2**	**65.4**

Blank cases are due to missing data. Abbreviations: LPG - lowest priced generic; OB - originator brand; HCT - hydrochlorothiazide; LI - low income; LMI - lower-middle income; UMI - upper middle income; HI - high income.

Across the cardiovascular medicines, captopril (LPG) had the lowest MPR in both the public and private sector. The highest MPR was found for hydrochlorothiazide. When comparing the public and private sector, it can be seen that for LPGs, the private sector was on average more expensive for all medicines. Importantly, countries that provide medicines for free in the public sector were not included to calculate the average MPR. For originator brand products, a direct comparison between the public and private sector could not be made due to the very limited availability of OB products in the public sector.

Lower income countries (LI and LMI) tended to have higher adjusted prices than the upper income countries (UMI and HI), both in the public and private sector. Again, some countries had consistently high or low prices across multiple medicines. High prices were found in El-Salvador and the Philippines, while low prices were often found in Fiji and Yemen.

### Brand premiums

**Figure 3:**
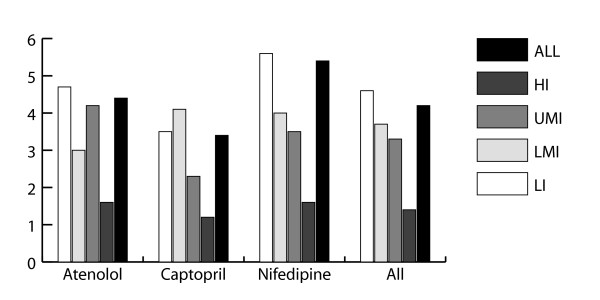
**Brand premiums in the private sector, by medicine and World Bank income group**. Abbreviations: LI - low income; LMI - lower-middle income; UMI - upper-middle income; HI - high income, Capt - captopril.

Brand premiums were calculated when possible; there was insufficient data available to calculate brand premiums in the public sector due to the low availability of OB products. Results are presented in figure 3. It was found that on average, brand premiums were lower in higher income countries as compared to lower income areas. Countries with high brand premiums for multiple medicines were Fiji and Peru, while low brand premiums could often be found in El-Salvador, Kuwait and the UAE.

### Affordability

Data for affordability were limited, in particular in the public sector; for losartan and nifedipine data were only available from two and three surveys, respectively. Therefore, the results may not always be representative of the entire sample.

In the public sector (n = 28, from 18 countries/regions), it cost on average 2.0 (LPG) and 8.3 (OB) day's wages to purchase one month of treatment with one of the cardiovascular medicines. On average affordability was better in the private sector (1.8 and 5.3 day's wages for the LPG and OB). When countries were matched, however, the private sector was usually less affordable than the public sector. Atenolol was most affordable, with an average of 1.1 day's wages for the LPG. When matched across surveys, the LPG was in all cases more affordable than the OB product, both in the public and private sector. Overall, cardiovascular medicines were least affordable in the low income regions. Upper-middle income countries scored particularly well, especially when compared to high income regions.

## Discussion

### Findings

Overall availability of the five cardiovascular medicines was low, with an average availability of 57.3% in the private sector and 26.3% in the public sector. However, availabilities of over 80% were observed in some countries (public and/or private sector). Availability of the lowest-priced generics was higher than originator brand products in most surveys and private sector availability was higher than public sector availability in all surveys. However, even in the private sector availability was not universal and prices were nearly always higher. In general, higher income countries had higher medicine availability than lower income countries, both in the public and private sector. Differences in the presence of CVD medicines on National Essential medicines lists, where they exist, may explain the (poor) availability of medicines in some countries. For example, in some countries enalapril was chosen as an essential ACE-inhibitor rather than captopril which was surveyed [23].

Average public sector procurement prices were relatively high; in the past, a MPR equal to or below 1.0 has been described as a reasonable procurement price [13]. Some countries achieved procurement prices well below the international reference price for a number of medicines, whereas others consistently paid higher prices [24]. As would be expected, generics were procured at lower prices than originator products. When comparing public sector procurement and patient prices, it was seen that in some cases patients were charged less than the procurement price. This could reflect that countries are aware of the high prices for essential chronic disease medicines and are using a cross-subsidizing system to lower these prices. In other cases however, patient prices in the public sector were much higher than procurement prices, which could be due to high mark-ups and taxes along the supply chain.

As with availability, patient prices varied greatly among surveys. In countries in which patients paid for medicines in the public sector, the average cost of LPGs was less than in the private sector for all medicines. However, a lack of availability in the public sector may force patients to purchase their medicines in the more highly priced private sector or forgo treatment altogether. For example, captopril had an adjusted MPR of 0.67 in the public sector in Jordan but only 61% availability. In the private sector, captopril had a much higher availability (90%), but the patient would need to pay 19.02 times the IRP.

As would be expected, generics were priced lower than originator brand products in nearly all surveys for all medicines. Our calculations of brand premiums show that to purchase an originator brand medicine costs 4.2 times as much as buying the lowest-priced generic. Interestingly, brand premiums were on average lower in upper-middle and high income countries. One would hope this smaller price differential was due to lower prices for the originator brand, but unfortunately this was due to higher prices being charged for generic products. This can occur when price regulations set the price for the generics discounted from the price charged for originator brand products, as occurs in Kuwait [13].

Overall, patient prices for cardiovascular medicines were high compared to international reference prices. For LPGs, the average adjusted MPR for the entire group of medicines was 15.5 in the public sector and 30.2 in the private sector.

The affordability data showed that on average one month of chronic treatment with one medicine for hypertension cost 1.8 day's wages. In all cases, originator brand products and the private sector were less affordable. Skipping one or two meals was often not enough to purchase treatment and often more than one day's wages were needed to purchase one month of treatment; therefore cardiovascular medicines may be labelled as unaffordable in a significant proportion of countries. Furthermore, an important part of the patient population requires combination therapy with multiple anti-hypertensives to reach treatment goals, amounting to an unaffordable treatment package. Finally, in one-income families, chronic treatment becomes particularly unaffordable if more than one family member has a medical condition that requires treatment.

### Strengths and weaknesses

The strength of this research lies in the standardized WHO/HAI methodology which has been validated through pilot and validation studies and adapted accordingly [17].

However, some difficulties do exist. Outcome measures, in particular availability, may be affected by the exclusion of alternate dosage forms and strengths or therapeutic alternatives. Also, availability data do not reflect average availability over time since availability data is only collected on one specific day. Furthermore, median price ratios may be skewed when international reference prices are based on limited data [18]. For this reason, price data for losartan were not analysed. It is important to realize that the MPR is a relative measure of price and does not provide information on the absolute price of a medicine; thus the absolute price of hydrochlorothiazide may be lower than that of nifedipine even though the latter has a lower MPR.

In some cases, data in one or more income group may have been skewed due to the small number of surveys in that category or dominance of one country within one income group. The latter occurred a number of times with surveys from India, which due to the unique nature of the Indian pharmaceutical market and the large number of Indian surveys, may have influenced outcomes in the low income group.

Calculating affordability based on the wage of the government worker may lead to an over-optimistic result since a significant proportion of the population earns less than this amount [14]. Alternative measures for affordability are currently under investigation [25]. A further problem for the affordability analysis was that only a limited number of surveys calculated affordability of these medicines.

### Implications

Based on the outcomes of this analysis, several implications for policy making can be emphasized. The poor availability in the public sector limits patient access to medical treatment by forcing them to resort to the more highly priced private sector, which has better, although not optimal, availability. The public sector is often seen as a last resort, but as our data show, the private sector fails to meet the needs of those least able to afford highly priced products. Improving public sector availability can be achieved by implementing more efficient procurement in the countries that pay high procurement prices, improving distribution systems and providing adequate and sustainable financing. Furthermore, focusing (limited) resources on a selected group of generic essential chronic disease medicines instead of aiming to supply a broad range of generic and originator brand medicines may lead to better availability of priority treatments [15].

In some countries, lowering procurement prices could help bring patient prices down [26]. Strategies to improve procurement efficiency include competitive procurement with price transparency, national pooled purchasing and purchasing by generic name [17]. Differential pricing based on the wealth of countries could also lower prices. This differential pricing already occurs for originator brand products to some degree, but not as much for generic products [27]. Promoting differential pricing could benefit lower income countries, not only affecting medicine prices, but also improving the availability of medicines. Options and strategies for differential pricing are currently being explored and promoted [28,29].

Addressing excessive mark-ups between procurement and patient prices could help bring prices down. For example in Syria, the maximum percentage mark-up allowed is determined based on the cost of the medicine; more expensive medicines are allotted a relatively lower mark-up to prevent preferential selling of highly priced products [15,30]. Furthermore, exempting medicines from tariffs and taxes, such as the value-added tax, will lower prices and prevent taxing of the sick [14,26]. It must then be ensured that savings obtained are passed on to patients. Furthermore, it has been found that in some countries, particularly in Sudan and China, revenue from medicine sales in the public sector is used to finance other parts of the public health care system [15,31,32]. Because the public sector is primarily used by the poor, this practice is inequitable and alternative sources of financing should be sought [15].

Furthermore, continuous support for the use of generic products can contribute to keeping the costs of medicines down, both in the public and private sector. Several strategies have been proposed, including ensuring product quality, encouraging or requiring generic substitution, preferential registration procedures and education of health care professionals and consumers [14].

Although generic medicines were usually priced lower and were more available than originator products in the private sector, some were still relatively overpriced as can be seen by the small brand premiums in high income countries [13]. In some cases, this could be prevented by implementing policies that do not regulate generic prices based on the price charged for brand products.

No matter how inexpensive medicines are in the private sector, the poorest sections of population in developing countries will still not be able to afford them. Further, chronic disease treatment requires lifelong therapy in order to prevent potentially life-threatening complications. Such demand is predictable and creates a responsibility on the health care system to ensure continued availability. Health insurance which covers out-patient chronic disease medicines is therefore of key importance. In this line of reasoning, investing in expensive facilities such as stroke or cardiac care units should only occur when reliable access to hypertensive medicines has been achieved.

## Conclusions

This analysis reveals that many problems remain with the availability, prices and affordability of cardiovascular medicines. To improve the situation, medicine policies should be adapted to promote the use of generic medicines, promote sustainable and reliable methods of procurement and financing and prevent excessive mark-ups in the supply chain. Also, continuing research into medicine pricing is necessary, as medicine prices continue to evolve and policy changes continue to have effects. Implementing medicine price monitoring programmes and performing price component analyses should be conducted to gain more insight into medicine prices and allow for adaptation of policies to specific countries.

## Competing interests

The authors declare that they have no competing interests.

## Authors' contributions

MvM carried out the data-analysis and drafted the manuscript. AC and RL conceived of the study and supervised its execution. ME and AC conceived and coordinated the secondary analysis data collection. All authors read and approved of the manuscript.

## Pre-publication history

The pre-publication history for this paper can be accessed here:

http://www.biomedcentral.com/1471-2261/10/25/prepub
